# HIV prevalence and risk behaviours among injecting drug users in six indonesian cities implications for future HIV prevention programs

**DOI:** 10.1186/1477-7517-9-37

**Published:** 2012-09-03

**Authors:** Guy Morineau, Liesbeth JM Bollen, Rizky Ika Syafitri, Nurjannah Nurjannah, Dyah Erti Mustikawati, Robert Magnani

**Affiliations:** 1 FHI Asia Pacific Regional Office, 19th floor, Tower 3, Sindhorn Building; 130-132, Wireless Road, Lumpini, Phatumwan, Bangkok, 10330, Thailand; 2 FHI Indonesia, Menara Salemba, Lantai 3; Jalan Salemba Raya No. 5, Jakarta, 10440, Indonesia; 3 Ministry of Health, Republic of Indonesia; Sub-Directorate for HIV/AIDS & STIs; Komplek Ditjen PP & PL Depkes RI, Jalan Percetakan Negara No. 29, Jakarta, 10560, Indonesia

**Keywords:** Injecting drug users, HIV, Indonesia, Harm reduction

## Abstract

**Background:**

The HIV prevalence among injecting drug users (IDUs) in Indonesia reached 50% in 2005. While drug use remains illegal in Indonesia, a needle and syringe program (NSP) was implemented in 2006.

**Methods:**

In 2007, an integrated behavioural and biological surveillance survey was conducted among IDUs in six cities. IDUs were selected via time-location sampling and respondent-driven sampling. A questionnaire was administered face-to-face. IDUs from four cities were tested for HIV, syphilis, gonorrhoea and chlamydia. Factors associated with HIV were assessed using generalized estimating equations. Risk for sexual transmission of HIV was assessed among HIV-positive IDUs.

**Results:**

Among 1,404 IDUs, 70% were daily injectors and 31% reported sharing needles in the past week. Most (76%) IDUs received injecting equipment from NSP in the prior week; 26% always carried a needle and those who didn’t, feared police arrest. STI prevalence was low (8%). HIV prevalence was 52%; 27% among IDUs injecting less than 1 year, 35% among those injecting for 1–3 years compared to 61% in long term injectors (p < 0.001). IDUs injecting for less than 3 years were more likely to have used clean needles in the past week compared to long term injectors (p < 0.001). HIV-positive status was associated with duration of injecting, ever been imprisoned and injecting in public parks. Among HIV-infected IDUs, consistent condom use last week with steady, casual and commercial sex partners was reported by 13%, 24% and 32%, respectively.

**Conclusions:**

Although NSP uptake has possibly reduced HIV transmission among injectors with shorter injection history, the prevalence of HIV among IDUs in Indonesia remains unacceptably high. Condom use is insufficient, which advocates for strengthening prevention of sexual transmission alongside harm reduction programs.

## Background

Injecting drug use drives HIV epidemics in several regions of the world [1,2]. HIV can spread rapidly among injecting drug users (IDUs) and can increase from virtually zero to HIV prevalence levels of 20-50% [3-5]. A study among IDUs in Bangkok, Thailand, showed an explosive spread of HIV with a prevalence of 1% in late 1987 to 43% in late 1988 [4].

Sharing of contaminated injecting equipment has driven the HIV epidemic in Indonesia [6]. Sentinel surveillance among IDUs in drug treatment programs in Jakarta indicated a rise in HIV prevalence from near zero in 1995 to over 50% in 2002 [7]. In 2006, the Ministry of Health (MOH) estimated that the 220,000 IDUs living in Indonesia contributed for 63% of all HIV infections , including 55% acquired through injecting and 8% sexually transmitted by infected IDUs [8].

The Indonesian government, including the National AIDS Commission, National Narcotic Board and Ministry of Health collaborates with international partners and local non-governmental organizations to implement harm reduction programs for IDUs since 2000 [8]. Community health centres (Puskesmas) and NGOs are providing harm reduction services, including distribution of clean needles and syringes through the Needle and Syringe Program (NSP) [9]. Peer educators provide bleach and information regarding HIV prevention and safe injecting practices. The methadone maintenance treatment program was initiated in 2003 by the WHO and the MOH at two pilot sites (Jakarta and Bali) and was scaled up to seven clinics serving approximately 1,000 clients by the end of 2006 [9]. A national prisons program was launched in 2005 by the Ministry of Justice and Human Rights with the aim of providing inmates with prevention, care and support services for tuberculosis and/or HIV [10]. However, drug use remains illegal in Indonesia resulting in challenges to implement the NSP.

Up to now information about HIV transmission risk among IDUs in Indonesia was limited to behavioural data. Data allowing assessing factors that drive the HIV epidemic are needed in order to inform programs and target those at highest risk. This paper provides behavioural and biological data about IDUs in Indonesia and discusses possible implications for future harm reduction programs.

## Methods

Behavioural survey data were collected from samples of 1,404 IDUs in six cities between August and November 2007: Bandung (West Java), Jakarta, Malang (East Java), Medan (North Sumatra), Semarang (Central Java) and Surabaya (East Java). IDUs interviewed in Bandung, Jakarta, Medan and Surabaya were asked to provide biological samples and were tested for HIV and syphilis (n = 992), and chlamydia and gonorrhoea (n = 728).

In Jakarta, Malang, Semarang and Medan, IDUs were selected through two-stage, time-location sampling. Lists of venues where IDUs congregated (streets, parks, private houses and drop-in) were developed by non-governmental organizations providing services to IDUs and local health authorities. Samples of venues were chosen via systematic-random sampling with probability proportional to venue size. All IDUs present at the time of data collection were selected for participation.

In Bandung and Surabaya, IDUs were recruited through respondent-driven sampling (RDS) as access to IDUs was insufficient to use time-location sampling. Eight male IDUs “seeds” were recruited purposively in each city, ensuring that they (1) lived in the city; (2) were aged 15–49; and (3) were part of an extended network of IDUs. All seeds and subsequent recruits were each given three coupons to recruit other IDUs. Recruiters received 40,000 Indonesian Rupiah (equivalent to USD 4, depending on exchange rates) for each recruit who could be verified to be an IDU and completed the survey interview. The survey was terminated when the target sample size of 250 was reached.

Survey field teams were drawn from staff of provincial offices of the Central Statistics Bureau and Provincial Health Offices; all received specialized training. Interviews were conducted in locations that offered visual and auditory privacy.

Interviewers obtained witnessed verbal consent and gathered behavioural data using a structured, pre-coded questionnaire. In four cities, a nurse then collected blood through finger prick, and in three cities participants provided self-collected, first-void urine. Behavioural and biological data were gathered anonymously and linked by identification numbers. Per MOH surveillance guidelines, participants received a coupon for free HIV counselling and testing at a nearby Community Health Centre and were given their participant number in order to access their STI test results and receive treatment free of charge if needed.

Blood specimens were collected in EDTA tubes, stored at 4-60C and transported to the nearest government laboratory to be tested for HIV and syphilis. HIV was tested using two parallel rapid tests: SD Bioline® HIV 1/2 3.0 (Standard Diagnostics, Suwon City, South Korea) and Determine® HIV-1 (Abbott, Abbott Park, IL, U.S.A.). Discrepant results were re-tested at the national research laboratory using two ELISA assays: Murex® (Murex Biotech, Dartford, United Kingdom) and Vironostika® (Biomérieux, Marcy l’Etoile, France). Syphilis was tested using a treponemal test – Determine Syphilis-TP® (Inverness Medical, Bedford, United Kingdom). Urine samples were tested for chlamydia and gonorrhoea by Cobas Amplicor® (Roche, Basel, Switzerland).

Behavioural data were double-entered using CSPro 2.6.007 (U. S. Census Bureau). Laboratory data were entered using Microsoft Excel. Analysis was performed using Stata 9.0 (Stata Corporation, College, Station, TX). Differences in frequencies were assessed using the Wald test, and means were tested with the Wilcoxon ranksum test. All tests were double sided and p-values <0.05 were considered significant. Background characteristics and behaviours were presented in term of frequency, mean and median. Associations between HIV and individual characteristics and/or behaviours were assessed using generalized estimating equations (GEE), which control for correlations within sampling “clusters.” Histograms representing the distribution of continuous variables that were tested in GEE were examined to ensure that they had a single mode and limited skewness. Independent contributions of factors to predicting HIV infection were assessed by fitting variables associated with HIV infection in bivariable analyses significant at the p ≤ 0.20 level into multivariable GEE models. Backward stepwise elimination was used and logits significant at the p < 0.05 level on Wald tests were retained in the final model. To accommodate readers more familiar with logistic regression, the logit results were converted to odds-ratios by taking their anti-logs.

Preliminary data analyses indicated that respondents sampled via RDS differed from those sampled via TLS with regard to key background characteristics – they were somewhat better educated and more likely to hold salaried positions, have resided in their interview city their entire life, and be somewhat longer-term drug injectors (all p < 0.01). However, because such differences can be controlled in multivariable analyses, we opted to use the full set of data available irrespective of sampling method. Analyses were performed on the multi-site pooled data assuming stratified cluster sampling, with drug injecting venues and RDS recruitment chains being considered as clusters.

The study protocol was approved by the Ethics Committee of the Indonesian Centre for Biomedical and Pharmaceutical Research, as well as the Protection of Human Subject Committee of Family Health International. Oral witnessed informed consent was obtained from participants for publication of the survey results.

## Results

### Background characteristics

A total of 1,404 IDUs participated in the survey with a median age of 27 years, 1350 (96.2%) were male. Most IDUs (63.6%) had attended senior high school, and 12.8% had attended college or university. Most (67.3%) had never married, 27.4% were currently married, and the remainder were separated, divorced or widowed. Most IDUs lived with parents and/or siblings (66.8%) and some lived with their spouse (20.1%). Forty percent of IDUs were independent workers, 26.6% held salaried positions, 12.7% were unemployed, and 7.5% were students. The vast majority of IDUs (81.2%) had lived their entire life in the city where they were interviewed.

### Injection experience and practices

Respondents began injecting drugs an average of 5.4 years prior to the survey interview (median = 5.0 years) at a mean age of 21.3 years (Table 1). Based upon self-reported injecting practices during the month prior to the survey, 70% of IDUs were considered regular injectors (at least one injection per day) and 30% were intermittent injectors.

**Table 1:** Reported behaviours among 1,404 Injecting Drug Users (IDUs) in six cities in Indonesia, 2007

**Behaviour**	**N**	**%**
Duration of injecting drugs		
< 1 year	33	2.4
1 - 3 years	481	34.5
≥ 3 years	879	63.1
Mean/Median		5.4/5.0
Age at first injection		
< 20 years	536	38.5
20 - 29 years	778	55.9
≥ 30 years	79	5.7
Mean/Median		21.3/21.0
Number of injections yesterday		
0	243	17.5
1	449	32.4
2	467	33.7
≥ 3	227	16.3
Mean/Median		1.6/2.0
Number of daily injections last month		
Don’t inject regularly	411	30.2
1	324	23.8
2 – 3	539	39.6
≥ 4	88	6.5
Number of people with whom shared last injection		
None	167	11.9
1	161	11.5
2	488	34.8
3 – 4	532	37.9
≥ 5	55	3.9
Mean/Median		2.0/2.3
Shared needle (borrowed or lent) last week	436	31.1
Shared drugs after mixing with water last week	934	70.4
Always carried needle last week	371	26.4
Used public needle last week	175	12.8
Injected with used needle at last injection	164	11.7
Cleaned used needle before injecting, at last injection	160	97.0
Injected in park past week	241	17.2
Injected in other city past year	516	36.9
Received needles from NSP last week	1060	75.5
Number of needles received from NSP last week		
Mean		4.8
Median		6.0
Had abscess at injecting site last year	904	66.3
Participated in rehabilitation program last year	680	48.6
Participated in methadone program last year	622	54.6
Oral methamphetamines last three months	863	61.5
Ever been in prison	417	29.7
Jailed for narcotic use last year	174	12.4
Ever tested for HIV	680	48.5
Tested for HIV last year	547	39.3
Took action to reduce risk of HIV infection last year	1014	72.5
Stopped using drugs	851	60.6
Reduced needle sharing	835	59.5
Reduced water sharing	858	61.1
Reduced number of fixes per day	932	66.4
Used bleach/disinfectant	815	58.1
Participated in NSP	911	64.9
Reduced number of injecting friends	832	59.3

Note. NSP: Needle Syringe Program.

Sixteen percent of IDUs reported sharing a needle (borrowed or lent) at last injection with a median of 2 persons. Twelve percent of IDUs injected with a previously used needle at last injection and thirteen percent used a public needle. The mean number of persons with whom respondents shared needles in the last week was 3.5 (95% CI 2.8 - 3.1) among IDUs injecting in parks compared with 2.9 (95% CI 3.2 - 4.1) among those injecting in other locations (p = 0.002). Nearly all injectors using previously used needles at last injection reported having cleaned the needle before injecting. Over 75% of respondents reported having obtained clean needles and syringes from an NSP in the prior week, receiving an average of five needles (median = 6.0). Twenty-six percent of IDUs always carried a needle in the last week; 907 (87.8%) of the 1,033 who did not carry a needle were afraid of being arrested by the police. Of the 1,404 IDUs, the majority (1,292) injected heroin in the last year, few injected amphetamines (74), methadone (15) and opium (22); 59.8% had used oral methamphetamines in the year prior to the survey. Around 50% of respondents reported having participated in detoxification programs in the last year and 55% in methadone programs.

One third (29.7%) of IDUs reported having ever been in prison and 8.3% of them injected for the first time while in prison. Of all survey respondents, 12.4% reported having been jailed for narcotic use last year. Of the 269 IDUs who were in prison last year, 139 (51.7%) received information on HIV and 26 (9.7%) received condoms while in prison.

### HIV and STI prevalence

**Figure 1:**
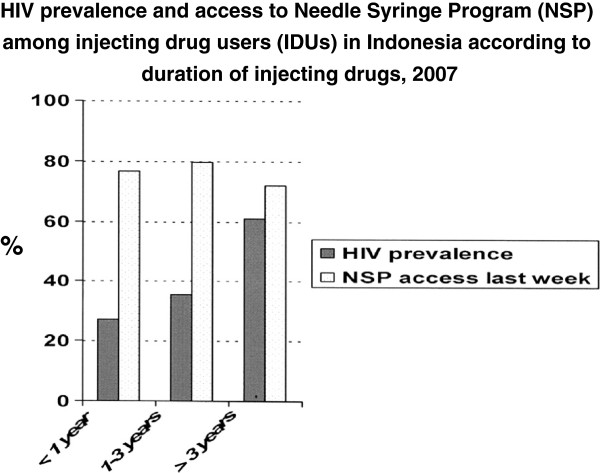
**HIV prevalence and access to Needle Syringe Program (NSP) among injecting drug users (IDUs) in Indonesia according to duration of injecting drugs, 2007**.

Estimated prevalence of HIV and STIs among IDUs in four cities are shown in Table 2. The prevalence of HIV was 52.4% (range 42.8- 56.0%). Prevalence of syphilis, gonorrhoea and chlamydia was low across cities. HIV prevalence was 27.0% among the 33 IDUs injecting for less than one year, 35.4% among those (n = 303) injecting for one to three years and 61.0% among those (n = 670) injecting for longer than three years (p < 0.001) (Figure 1). In addition, a higher proportion of injectors with shorter injection history accessed NSP in the last week compared with those injecting for longer duration (76.5%, 79.5% and 71.9%, respectively; p = 0.003) (Figure 1).

**Table 2:** Prevalence of HIV and Sexually Transmitted Infections (STIs) among Intravenous Drug Users (IDUs) in four cities in Indonesia, 2007

**City**	**HIV %(n/N)**	**Syphilis %(n/N)**	**Chlamydia %(n/N)**	**Gonorrhoea %(n/N)**	**Chlamydia/gonorrhoea % (n/N)**	**Any STI %(n/N)**
Medan	55.6% (139/250)	2.4% (6/250)	5.3% (13/246)	0.0% (0/249)	5.3% (13/249)	7.2% (18/249)
Jakarta	55.0% (137/249)	0.8% (2/242)	6.0% (14/234)	1.3% (3/234)	7.3% (17/234)	8.1% (19/234)
Bandung	42.8% (107/250)	0.0% (0/250)	NA	NA	NA	NA
Surabaya	56.0% (140/250)	1.6% (4/250)	5.72% (14/249)	(1.2%) (3/249)	6.8% (17/249)	8.4% (21/249)
All cities	52.4% (523/999)	1.2% (12/992)	5.6% (41/729)	0.8% (6/732)	6.4% (47/732)	7.9% (58/732)

NA = Not available.

### Factors associated with HIV infection

Duration of injecting was associated with a higher likelihood of HIV infection, with the odds of infection increasing by 12% for each year of injecting (Table 3). Respondents who injected in a park in the previous month were significantly more likely to have tested HIV-positive (AOR = 1.64), as were those who had been jailed (AOR = 1.58). IDUs who tested positive for gonorrhea or chlamydia were less likely to have been infected (AOR = 0.51). However, infection with chlamydia and/or gonorrhoea was neither associated with variables on types and numbers of sexual partners in the past month, nor with reported condom use. Unprotected commercial sex in the last month was not associated with higher risk for HIV infection.

**Table 3:** Bivariate and multivariate associations between selected factors and HIV Infection among 729 Injecting Drug Users (IDUs) in four cities in Indonesia, 2007

**Factors**	**Bivariate Analysis**			**Multivariate Analysis**		
	**Odds Ratio**	**95% CI**	**p-value**	**Adjusted Odds Ratio**	**95% CI**	**p- value**
Age (continuous)	1.07	1.04 – 1.11	<0.001			
Duration injecting drugs (continuous)	1.16	1.12 - 1.21	<0.001	1.14	1.09 - 1.20	<0.001
No. injections past week (continuous)	1.02	0.89 - 1.18	0.300			
No. people who shared injection with last week	0.97	0.93 – 1.02	0.254			
Shared needles in last week *	1.16	0.82 – 1.47	0.537			
Shared drugs after mixing with water last week *	1.16	0.72 – 1.29	0.803			
Injected in park last week *	1.19	1.24 - 2.44	0.001	1.64	1.11 - 2.42	0.012
Received needles from NSP last week *	0.86	0.60 - 1.24	0.426			
Participated in methadone program last year *	1.43	1.05 - 1.94	0.022			
Participated in rehabilitation program last year *	1.41	1.09 - 1.82	0.009			
Used oral methamphetamines last 3 months*	0.93	0.76 - 1.13	0.457			
Ever reached by outreach worker *	1.44	1.05 - 1.96	0.023			
Ever been jailed *	1.86	1.43 - 2.42	<0.001	1.58	1.13 - 2.21	0.007
Had unprotected commercial sex past month	0.82	0.59 - 1.14	0.233			
Ever tested for HIV *	1.57	1.20 - 2.01	0.001			
Tested positive for gonorrhoea and/or chlamydia*	0.53	0.29 - 0.96	0.036	0.51	0.27 - 0.94	0.032
Tested positive for syphilis*	1.51	0.48 - 4.70	0.477			

* For indicated variables, “no” is reference category with OR = 1.0 (not shown).

Note. NSP; Needle Syringe Program.

### Indicators for HIV transmission

Of the 523 HIV-positive IDUs, 373 (71.3%) had sex in the year prior to the survey (Table 4). Half of the HIV-positive IDUs had a female steady partner and 14.8% reported to use condoms consistently. Also, consistent condom use was rare among the 185 HIV-positive IDUs who had female casual partners (21.6%) and among the 105 who had commercial partners (27.9%). Twenty-two percent of the 523 HIV-positive IDUs reported to have given their used needle to another IDU in the last week.

**Table 4:** Indicators for HIV transmission among 523 HIV-positive Injecting Drug Users (IDUs) in four cities in Indonesia, 2007

**Behavior**	**HIV positives**
Had sex past year	71.3
Had steady partner past year	50.3
Used condoms with steady partner past month	
Never	47.7
Seldom	23.9
Often	13.6
Always	14.8
Had sex with casual female partner past year	35.4
Used condoms with casual female partners past month	
Never	42.3
Seldom	22.5
Often	13.5
Always	21.6
Sold sex last year	2.7
Purchased sex last year	20.0
Condom use with FSW last month	
Never	45.2
Seldom	20.2
Often	6.7
Always	27.9
Gave used needle to other IDU last week	21.1

Note. FSW; female sex worker.

## Discussion

HIV prevalence among community-based IDUs in four major Indonesian cities was high (53%) in November 2007. The HIV epidemic may have slowed down somewhat as injectors with shorter injection history had a lower HIV prevalence and a higher proportion were accessing clean needles from the NSP than those injecting for a longer duration. Overall, 70% of IDUs reported having taken some action to reduce HIV transmission risk.

Implementation of NSP should be highly successful in controlling the HIV epidemic among IDUs as demonstrated in a recent publication from Australia [11]. However, drug use remains illegal in Indonesia and subsequent law enforcement is conflicting with implementation and support for NSP. Indeed, a quarter of IDUs in our survey reported to have been imprisoned because of drug use. Although most received needles from NSP, the main reason reported for not always carrying a needle was the fear of police arrest. The distribution of needles and syringes is currently shifting from local NGOs to community health centres (Puskesmas), which may affect the uptake of services.

Imprisonment was an independent risk factor for HIV infection. New inmates could be HIV-positive at the time of incarceration or they may get infected while in prison as injecting drugs seems to continue in prison under unsafe conditions. Although Indonesia implemented a national strategy for HIV prevention in prisons [10], challenges remain to implement appropriate HIV prevention and care services in this setting.

Injecting in a park was another risk factor for HIV infection. It is well established that the size and density of social and injecting networks are key factors in the transmission of HIV among IDUs [12,13]. Indeed, a recent study in Pakistan pointed out large sharing networks as a key factor for explosive HIV prevalence growth among IDUs [14]. Assuming that IDUs who recently injected in parks did so in the past as well, the high prevalence of HIV found among those injecting in parks plausibly reflects their exposure to larger and more diverse needle sharing networks than those not injecting in parks.

Sexual risk behaviour, such as unprotected commercial sex, was not associated with HIV, indicating that the main route for HIV acquisition among IDUs is unsafe injections rather than sexual intercourse. Surprisingly, infection with gonorrhoea or chlamydia (NG/CT) was associated with lower likelihood of HIV infection, but our data could not identify any behaviours associated with both HIV infection and NG/CT.

Around half of IDUs were HIV-positive with high potential of onward HIV transmission. Sharing used needles by HIV-positive IDUs was common as well as unprotected sex with steady, casual and commercial partners. Condom use was especially rare with steady partners, which is concerning as steady partners will have multiple exposures to unprotected sex. It was pointed out earlier that sexual behaviour among IDUs in Indonesia has the potential for HIV spread to non-injectors [15]. Condom use promotion and provision should be strengthened alongside NSP programs together with disclosure counselling, reaching out and promotion of HIV-testing for partners. The high prevalence of HIV also emphasizes the need to assess and ensure access to antiretroviral treatment (ART).

This survey presents several limitations. As it is the case for all cross-sectional studies, it is not possible to know if the risk factors associated with HIV pre-existed the time of infection. The pooling of population sampled with different methodologies threatens the validity of the statistical tests in bivariable analysis. However, this heterogeneity was somehow addressed in the regression analysis using GEE. Syphilis testing did not include a quantitative RPR test; hence syphilis test results included both active syphilis and serologic scars.

Although the uptake of NSP has possibly reduced HIV transmission among injectors with shorter injection history, the prevalence of HIV among IDUs in four Indonesian cities remains unacceptably high. Sharing of injecting equipment is sustained by the fear of police arrests when carrying needles. Shifting emphasis from a security approach to a public health approach would contribute to reducing HIV transmission among IDUs in Indonesia. Sexual partners of IDUs are at high risk for getting infected with HIV and programmes should include strategies to this vulnerable population.

## Competing interests

The authors declare that they have no competing interest.

## Authors’ contribution

GM, LB and RM drafted the paper. GM conducted data analysis. GM, RM and DEM designed the survey and overviewed its implementation. RIS and NN provided technical review for survey design and reviewed the paper. All authors read and approved the final manuscript.
